# The IMAP Magnetometer

**DOI:** 10.1007/s11214-026-01277-8

**Published:** 2026-05-04

**Authors:** T. S. Horbury, H. L. O’Brien, C. Greenaway, A. Roberts, A. Crabtree, M. Tomes, M. Facchinelli, M. Finlayson, M. Bharatia, E. Fauchon-Jones, M. Tapley, S. Pope, D. Jones, I. Richter, S. Dalla, C. Russell, N. A. Schwadron, M. Gkioulidou, D. J. McComas

**Affiliations:** 1 Imperial College London, London, SW7 2AZ UK; 2 University of Oxford, Oxford, UK; 3 Southwest Research Institute, San Antonio, TX USA; 4 Applied Physics Laboratory, Johns Hopkins University, Laurel, MD USA; 5 Technical University of Braunschweig, Braunschweig, Germany; 6 University of Lancashire, Preston, UK; 7 Institute of Geophysics and Planetary Physics / UCLA, Los Angeles, CA USA; 8 University of New Hampshire, Durham, HN, USA; 9 Department of Astrophysical Sciences, Princeton University, Princeton, NJ USA

## Abstract

The magnetometer (MAG) is one of the ten scientific instruments on the Interstellar Mapping and Acceleration Probe (IMAP), which will take in situ and remote measurements from a Sun-Earth L1 halo orbit. MAG contributes to IMAP science goals of investigating the acceleration and propagation of energetic particles, as well as providing real-time space weather monitoring data. The magnetometer is a conventional dual sensor fluxgate instrument with a noise floor under 10 pT at 1 Hz, taking science measurements continuously at 2 vectors/s as well as a burst mode of 64 vectors/s for at least 8 hours per day. It also provides a real-time space weather monitoring product at 4 second cadence. We describe the requirements, design and performance of the instrument, including a novel lossless compression algorithm. Data products, processing and calibration plans are presented.

## Introduction

The IMAP magnetometer, MAG, is a high precision dual fluxgate instrument which measures the magnetic field in the vicinity of the spacecraft from the timescales beyond a solar rotation down to less than 10 ms, well below the proton gyroscale. Knowledge of the magnetic field and its fluctuations is essential to IMAP’s science goals (McComas et al. 2025), which include the understanding of the acceleration and propagation of pick-up ions and other energetic particles in the solar wind. It is also central to IMAP’s real-time space weather forecasting capability (Lee et al. 2025) and, in combination with magnetic field measurements from other spacecraft in the L1 halo orbit, provides unprecedented information on the meso-scale structure of the solar wind and transient structures such as shocks and coronal mass ejections.

The IMAP magnetometer is of a different design than originally proposed for the mission (McComas et al. 2018) and has been provided by Imperial College London using funding from the UK Space Agency. Building on heritage from the Solar Orbiter magnetometer (Horbury et al. 2020) which has over 5 years of successful in-flight operations, it satisfies and in many cases greatly exceeds the IMAP magnetic field measurement requirements. It also implements an innovative lossless compression algorithm to approximately double the data return compared to the baseline.

This paper briefly describes the scientific context of MAG within the overall IMAP payload and how this drives the instrument requirements. The instrument design is described in detail including key developments and changes compared to the Solar Orbiter instrument. Its performance is presented, along with existing ground calibrations and the planned in-flight calibration procedures. The work to ensure overall spacecraft-level magnetic cleanliness and its verification is also discussed. MAG operations are by design as simple as possible and these are described along with the expected data products.

## Science Requirements

The IMAP mission (McComas et al. 2025) is the fifth in NASA’s Solar-Terrestrial Probes line with objectives to improve our understanding of the local interstellar medium, its interaction with the solar wind and heliospheric magnetic field, and the processes by which particles are accelerated near the Sun and elsewhere. To achieve these goals it carries a broad payload of 10 science instruments. Five instruments remotely characterise the heliosphere-interstellar medium interaction through energetic neutral atoms (ENAs from 5 eV to 300 keV: IMAP-Lo, IMAP-Hi, IMAP-Ultra), dust (IDEX) and UV radiation (GLOWS). The other five instruments measure solar wind electrons (SWE: 1 eV–5 keV) and ions at thermal (SWAPI: 0.1–20 keV/q), suprathermal (CoDICE: 0.5–80 keV/q and 0.05–2 MeV/nuc) and higher energies (HIT: 2–70 MeV/nuc) as well as the local magnetic field (MAG). A particular goal of the in situ part of the payload is the study of the acceleration of ions, and especially interstellar pick-up ions, around interplanetary shocks (Cohen et al. 2026) since these play a key role in the production of outer heliospheric ENAs.

The magnetic field is a vital parameter in space plasmas: it controls the propagation of ions and electrons; the energy it carries can energise particles through collisionless shocks, reconnection and turbulence processes; and field-particle interactions can scatter and thermalise non-Maxwellian distributions (e.g. Verscharen et al. 2019). As a vector that can be measured with high precision it characterises fine scale plasma phenomena such as shocks and instabilities as well as the large scale structure of the interplanetary medium.

The science goals of the IMAP mission require a high quality measurement of the magnetic field local to the spacecraft, with sufficient precision and cadence to detect and characterise all the phenomena described above. From a science perspective, measurements are required with a resolution and cadence fine enough to resolve fluctuations from the largest scales to the ion gyroscale and a precision good enough accurately to characterise the magnetic field vector and its variations over these scales. Given typical properties of the interplanetary magnetic field at L1, these have been quantified as the set of requirements listed in Table 1.

**Table 1:** IMAP MAG requirements and instrument performance

Parameter	Requirement	IMAP MAG performance
Range	±120 nT	±512 nT in most sensitive range
Resolution	<100 pT	4.5 pT in most sensitive range
Minimum sampling rate	2 vectors/s	2 vector/s
Maximum sampling rate	128 vectors/s	128 vectors/s, will normally operate burst mode at 64 vectors/s
Burst mode coverage	15 minutes/day	At least 8 hours/day

Four second MAG data will also be relayed in real time, via the IMAP Active Link for Real-Time Data (I-ALiRT: see Lee et al. 2025) system, for prompt space weather forecasting.

Of particular scientific interest is the combination of data from IMAP MAG with those of other spacecraft in the L1 halo orbit, such as Wind (Lepping et al. 1995), ACE (Smith et al. 1998), DSCOVR, Aditya-L1 (Yadav et al. 2025) and SWFO-L1 (Torbert et al. 2026), the latter of which is launching with the IMAP launch vehicle. While it is not clear how many spacecraft will be operating at L1 during the IMAP mission, the combination of data from those available will make it possible to study the 3D structure of the magnetic field and solar wind plasma, as well as for example shock rippling and variability (e.g. Trotta et al. 2023), on separations around the solar wind correlation scale (e.g. Weygand et al. 2011) which are otherwise not accessible.

Achieving its science requirements puts constraints not only on the MAG instrument, but also the IMAP spacecraft and the procedures by which the data are processed and calibrated. In this paper we describe the relevant aspects of the instrument, spacecraft and ground segment which together will produce science quality magnetic field measurements for the IMAP mission.

## Instrument Design and Qualification

**Fig. 1:**
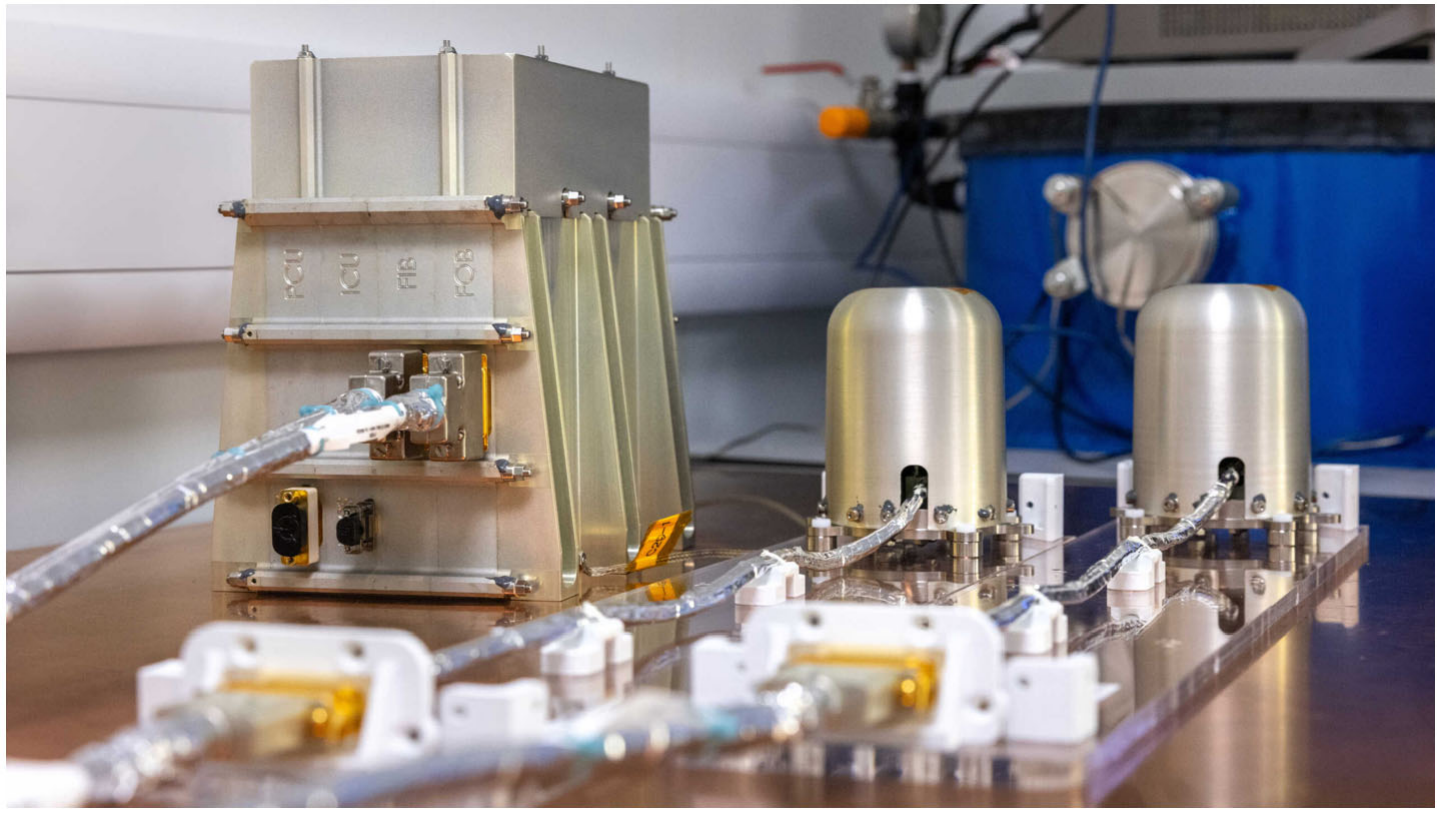
The IMAP flight magnetometer, with two sensors on the right and the electronics box on the left. Image credit: Thomas Angus, Imperial College London

The IMAP magnetometer (Fig. 1) is a relatively conventional dual fluxgate design. Given the short time available – the flight model was integrated onto the spacecraft less than 3 years from first funding – and in accordance with the overall IMAP development approach, the decision was made to minimise development work by maximising use of the successful Solar Orbiter magnetometer design. Key changes include replacement of the SpaceWire data interface with UART and removal of unnecessary sensor heaters but many small changes, some of which were unanticipated, also proved necessary.

**Fig. 2:**
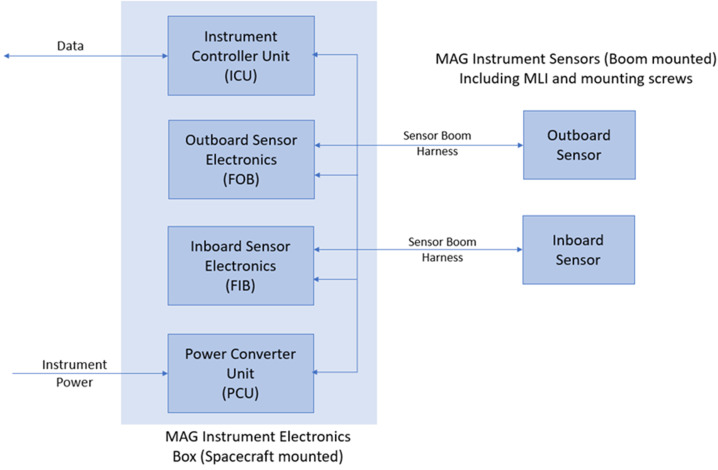
MAG block diagram

Key elements of the magnetometer design (see Fig. 2) are: two sensors, located on the MAG boom; an electronics box (ELB) accommodated within the spacecraft body near the boom root; and harness connecting the sensors to the ELB. The ELB contains a power supply board (PCU), one front-end electronics (FEE) board for each sensor, and one board carrying the instrument controller unit (ICU) which communicates with each FEE and with the spacecraft. Key MAG parameters are shown in Table 2.

**Table 2:** IMAP MAG summary

Parameter	Value
Instrument Type	Dual sensor fluxgate magnetometer
Normal mode cadence	2 vectors/s from both sensors
Burst mode cadence	64 vectors/s outboard sensor; 8 vectors/s inboard
Burst mode coverage	>8 hours/day
Digital resolution	4.5 pT
Noise at 1 Hz	<10 pT
Nominal range	±512 nT at 4.5 pT resolution
Maximum range	±60,000 nT at 450 pT resolution
Mass	5 kg
Power	7.75 W
Telemetry	563 bps normal mode; 13,079 bps burst mode

### Principle of Operation

Fluxgate magnetometers are a long-established technology for high precision and stability vector measurement of magnetic fields in both terrestrial and space applications (e.g. Acuña 2002). At its simplest, a fluxgate magnetometer uses a ferrous core about which is wound a wire through which a drive signal at a frequency $f$ is driven, forcing the core into periodic saturation. If an ambient magnetic field is present then the core saturates more rapidly when driving in the sense with that ambient field, and more slowly when driving against it, resulting in an asymmetric saturation which can be detected with an additional sense winding as a hysteresis signal at 2$f$. In practice, in order to improve linearity and reduce noise, instruments can then impose a feedback current to null out the ambient field: it is the amplitude of this current which is then proportional to the ambient magnetic field. Multiple cores can be used, with orthogonal windings, to measure the vector field.

The fluxgate magnetometer principle predates the Space Age and indeed digital electronics, but recent designs incorporate digital aspects: implementations differ and for IMAP, like Solar Orbiter, the sense signal is digitised and the feedback current generated by a digital to analogue converter (DAC).

### Sensors

The two MAG sensors, one outboard and one inboard, are known as MAGo and MAGi respectively and are functionally identical. The mechanical design is identical to Solar Orbiter: each sensor comprises two magnetically soft cores procured from Ultra Maritime, mounted in a ceramic Macor block to maintain their orthogonal orientation. Each core has twin, orthogonal windings and so can measure the field in two directions: the X sensor axis is sensed by both cores with the sense windings in series. Two small PCBs hold tuning capacitors, thermistors and connections between the cores and the harness: the heaters used on Solar Orbiter have been removed because the thermal environment for IMAP, with constant solar illumination onto the boom, means that the sensors will stay well within their qualified range of −45 °C to +59 °C during routine operations. The Macor block is in turn held in a Tufnol standoff to increase thermal isolation of the sensor, which is mounted to a titanium baseplate. This baseplate is then mounted to a matching plate on the carbon fibre boom. The sensor is protected by a spun aluminium cover and covered by multi-later insulation (MLI) in flight.

**Fig. 3:**
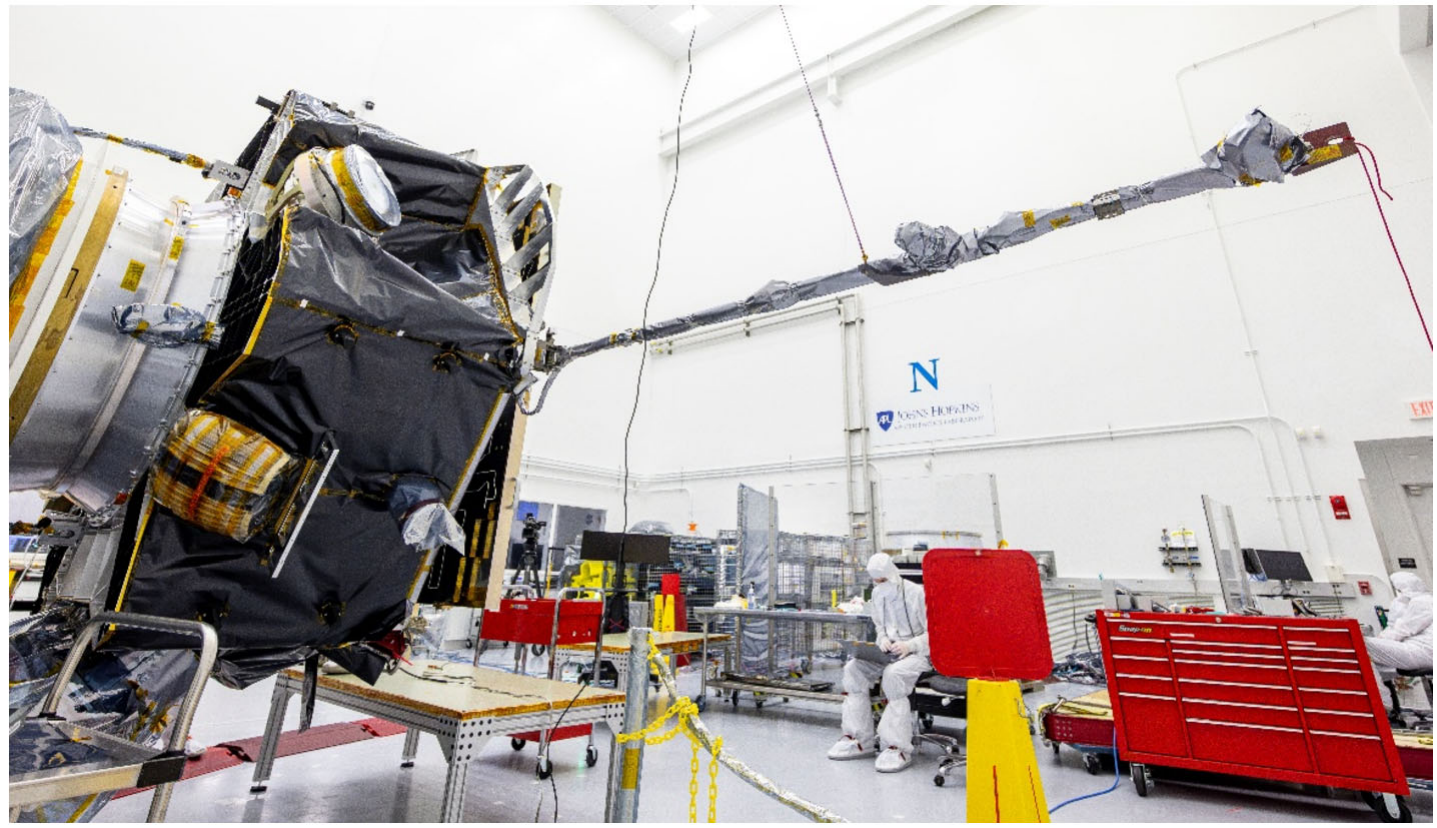
The IMAP spacecraft with the MAG boom extended during a deployment test: the two MAG sensors are visible, covered in MLI. Credit: NASA/Johns Hopkins/Princeton/Ed Whitman

The MAG boom is a 2.5 m single hinge, rigid carbon fibre tube, deployed using a frangibolt: MAGo is accommodated at the tip, while MAGi is 75 cm inboard (Fig. 3). As well as providing redundancy in case of failure, this dual sensor configuration makes it possible to use “gradiometer” methods (e.g. Ness 1971) to quantify and remove spacecraft-generated fields, which will be especially important for the I-ALiRT space weather products as described below.

### Front End Electronics

**Fig. 4:**
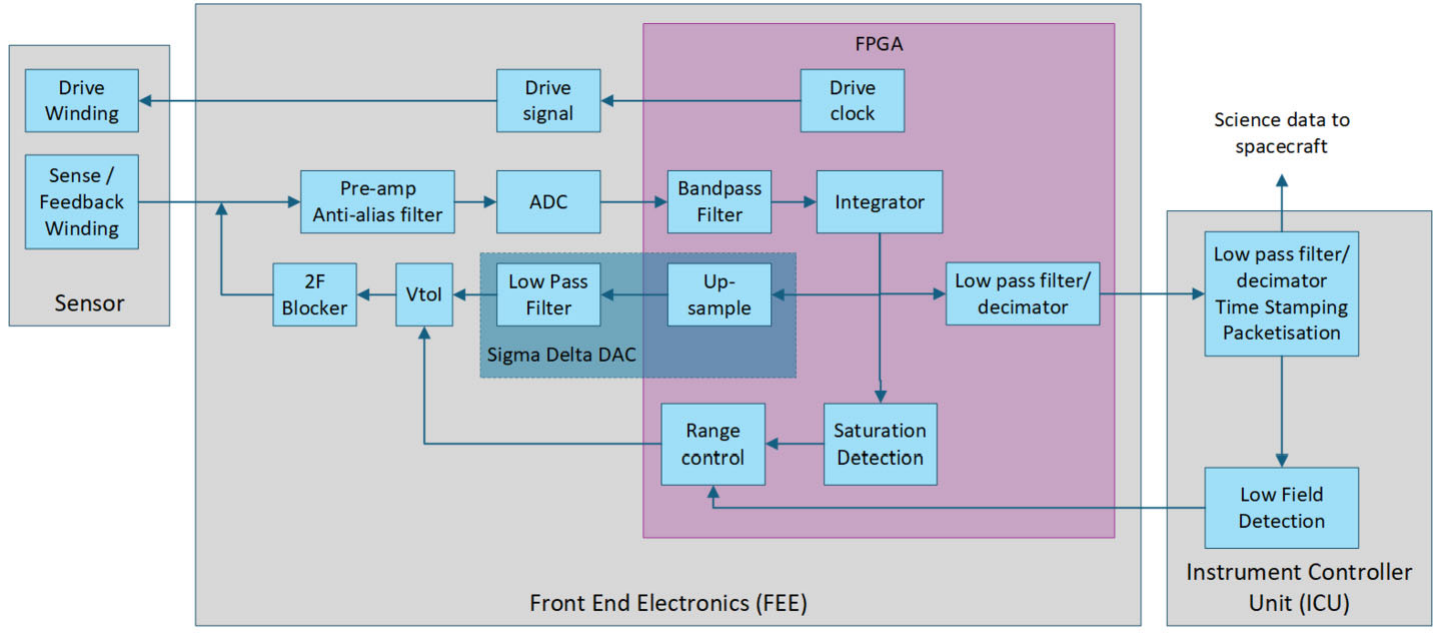
MAG instrument schematic. In practice there are two sensors, each with one FEE board, communicating with the single ICU board

The FEE design (Fig. 4) is functionally identical to that on Solar Orbiter. The drive signal is at $f=15.36$ kHz and the sense signals (one per axis) are detected at $2f$ using a 14 bit analogue to digital converter (ADC), which samples 64 points across the drive period. A RTAX FPGA applies a bandpass filter to this signal to quantify the open loop field value, which is fed into an integrator to operate in closed loop. The output of the integrator is proportional to the field applied and is fed into a sigma-delta digital to analogue converter (DAC) within the FPGA. The DAC incorporates an up-sampled 250 kHz single bit data stream and a downstream analogue, third order low pass Butterworth filter with a 500 Hz cutoff frequency. This is then passed to a voltage to current converter before being applied to the feedback coil. The integrator output is also passed through a third order low pass cascaded integration comb (CIC) filter to generate the digital output field value, which is transferred to the ICU at 1920 vectors/s via SPI link.

In practice, the Solar Orbiter FEE design has worked flawlessly and displays very low noise in flight. It required no changes for IMAP.

### Instrument Control Unit

The ICU is based around a RTAX2000S/SL FPGA on which is programmed a fault tolerant 25 MHz Leon3FT-RTAX PC1 provided by Aeroflex Gaisler, an identical configuration to Solar Orbiter. This includes several additional units including two SPI cores to communicate with the FEE boards and two Space Wire cores which are not needed for IMAP, but retained to reduce development complexity: instead a UART interface has been implemented for communication with the spacecraft. Solar Orbiter used EEPROM memory but the physical size of the components made them hard to solder and pass vibration qualification, so for IMAP these were replaced with one 2M Magnetic Random Access Memory (MRAM) flash module which has a more compact footprint.

The ICU contains three types of memory: first, a Programmable Read Only Memory module which contains a copy of the MAG boot software (BSW) and cannot be changed in flight. The MRAM holds three copies of the application software (ASW), as well as the Instrument Configuration Vector (ICV): the MRAM can be altered in flight to allow for software updates if necessary or changes to the default instrument behaviour or FDIR limits. The boot software can be commanded to transition into any of the three app software slots. Finally, the ICU contains a single 1Mx39 volatile Static Random Access Memory (SRAM) unit: 32 bits are used for data with 7 bits used for Error Detection and Correction (EDAC), which is managed natively by the Leon3FT-RTAX.

**Fig. 5:**
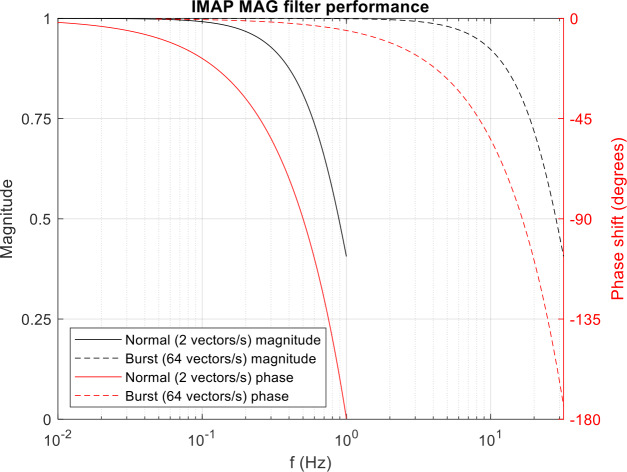
MAG low pass filter passband characteristics (relative magnitude of the filtered signal in black; phase shift of the original signal in red) for the 2 vectors/s normal mode data (solid line) and 64 vectors/s burst mode data (dashed line)

The Leon3FT-RTAX runs a modified version of the RTEMS software for both BSW and ASW, with only core functionality retained in BSW; memory updates can be performed from either. The ASW operates the instrument during science operations including: communication with the spacecraft; responding to commands; monitoring instrument health; decimating, filtering and time-stamping data from the FEE boards to provide science data streams at the required cadence; and generation of I-ALiRT data packets for real-time space weather monitoring. The ICU implements a second order CIC low pass filter-decimator (e.g. Hogenauer et al. 1981: filter characteristics shown in Fig. 5) to produce the required output data rate from the incoming 1920 Hz data streams from the FEE.

A significant software development task was the transition from SpaceWire to UART for the data interface. Maximising reuse of Solar Orbiter design resulted in a 1 byte UART buffer, which drives CPU load and required careful optimisation of task priorities to ensure reliable operation. The software has proved to be very stable under testing: the Solar Orbiter flight software, from which it has been developed, has not exhibited a bug after over 5 years in flight and has operated for over 18 months continually without issue.

In order to maximise science return within the telemetry budget, a novel lossless compression scheme has been implemented; a very similar scheme was developed in parallel for Solar Orbiter MAG and has been running successfully in flight since November 2024. In this scheme data is compressed in each packet, with the first vector recorded in full and subsequent vectors enclosed as differences for each component. The differences within one packet are typically small compared to the instrument range, so this results in values with only a small number of non-zero bits. These are then encoded using zig-zag encoding (to allow for negative numbers but still keep them small) and Fibonacci compression. The latter takes advantage of Zeckendorff’s theorem (e.g. Graham et al. 1994), that every positive integer can be represented as a unique sequence of non-consecutive Fibonacci numbers. To our knowledge this compression method has not previously been used with spacecraft measurements. Given the wide applicability of the algorithm for such data it will be presented in detail in a subsequent paper, but it has several positive qualities. It results in compression which is lossless and never generates packets larger than uncompressed data, although the ultimate packet size is not predictable. It also has almost negligible CPU overhead, particularly for IMAP MAG where the small increase in processor overhead is offset by the reduced load in making and transmitting smaller packets. On Solar Orbiter, where the compression has run flawlessly for many months, compression ratios near 3 are routinely achieved. Combined with optimisation of MAG packet structure, we expect to record at least 8 hours of burst mode data each day, compared to a baseline requirement of 15 minutes, and hence dramatically improve the probability of capturing the internal structure of interplanetary shocks for comparison with SWAPI, CoDICE, HIT and SWE measurements.

Other software developments from the Solar Orbiter baseline included generation of I-ALiRT packets and the elimination of the normal mode data stream when burst mode data is generated, to reduce the overall telemetry budget.

### Power Converter Unit

The PCU takes spacecraft power at a nominal 31 V and uses a switch mode DC-DC converter operating at 131.072 kHz to provide the range of required voltages to the ICU and FEE boards: ±8.5 V, +3.3 V, +2.5 V, 1.8 V and +1.5 V. The design incorporates aspects of the Solar Orbiter and JUICE PCU boards. Since sensor heaters have been removed for IMAP, the heater circuitry has also been removed from the PCU.

### Qualification

A MAG Engineering Model, representative in “fit, form and function” but with not all parts of flight quality, was built to verify the design and performance, including extensive qualification testing, following which the Flight Model was constructed. The FM has undergone a standard proto-flight qualification flow, including vibration and thermal vacuum testing at Rutherford Appleton Laboratory in the UK. Sensor and electronics box mechanical and thermal test specifications are listed in Table 3, Table 4 and Table 5. EMC testing at Airbus DS, Toulouse identified some radiated susceptibility which manifested in spurious sensor temperature readings and some variations in the apparent field offsets. This was identified as likely being due to the boom harness. Additional grounding was added to the boom harness as a risk mitigation. Follow-up testing was done with the FM at APL, where combined with refined estimates of the expected EMC performance, it was determined that there will be no impact on science or housekeeping data from the instrument in flight.

**Table 3:** Sensor mechanical test specifications

Shock specification		Sine vibration test				Random vibration test	
All axes		Perpendicular to mounting interface		Parallel to mounting interface		All axes	
Frequency (Hz)	Peak acceleration (g)	Frequency (Hz)	Peak acceleration (g)	Frequency (Hz)	Peak acceleration (g)	Frequency (Hz)	ASD level (g^2^/Hz)
0.100	40	5	0.63	5	0.63	20	0.026
2335	1430	35	31	30	22	20–50	+6 dB/oct
10,000	1430	39	31	34	22	50–800	0.16
		46	1.25	41	1.25	800–2000	−6 dB/oct
		100	1.25	100	1.25	2000	0.026
						Overall	14.1 G_rms_

**Table 4:** Electronics box mechanical test specifications

Sine vibration test				Random vibration test	
Perpendicular to mounting interface		Parallel to mounting interface		All axes	
Frequency (Hz)	Peak acceleration (g)	Frequency (Hz)	Peak acceleration (g)	Frequency (Hz)	ASD level (g^2^/Hz)
5	0.63	5	0.63	20	0.0063
19	9	22	12	20–80	+6 dB/oct
23	9	26	12	80	0.1
30	1.25	33	1.25	300	0.1
100	1.25	100	1.25	300–450	−6 dB/oct
				450	0.045
				550	0.045
				550–700	+6 dB/oct
				700	0.16
				800	0.16
				800–2000	−6 dB/oct
				2000	0.026
				Overall	12.1 G_rms_

**Table 5:** IMAP MAG flight model pre-launch thermal cycling

	Electronics box		Sensors	
	Operational	Non-operational	Operational	Non-operational
Hot cycles	6 cycles to 50 °C	1 cycle to 60 °C	6 cycles to 59 °C	Bake out to 90 °C
		Bake out to 90 °C		
Cold cycles	6 cycles to −25 °C	1 cycle to −30 °C	5 cycles to −55 °C	1 cycle to −55 °C
			1 cycle to −55 °C in low field	

The FM unit, following integration onto the spacecraft, has taken part in the observatory-level environmental test flow, including extended thermal vacuum testing.

MAG has performed well throughout the test campaigns and is ready for flight.

## Instrument Performance

Testing on the Engineering Model instrument confirmed comparable performance to that of the Solar Orbiter Flight Model, and the IMAP Flight Model has also shown excellent performance.

**Fig. 6:**
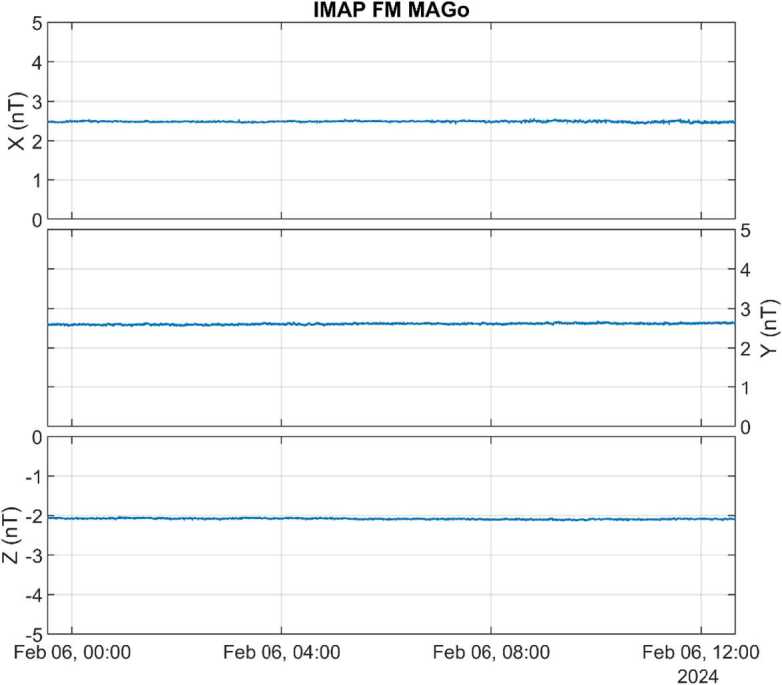
Time series of MAGo flight model data in sensor coordinates recorded inside a magnetic shielding can at Imperial College London

The instrument exhibits very low noise, and high stability. Demonstrating stability is challenging in central London, but Fig. 6 shows an example overnight run in a magnetic shielding can, where stability better than 0.1 nT is clear.

**Fig. 7:**
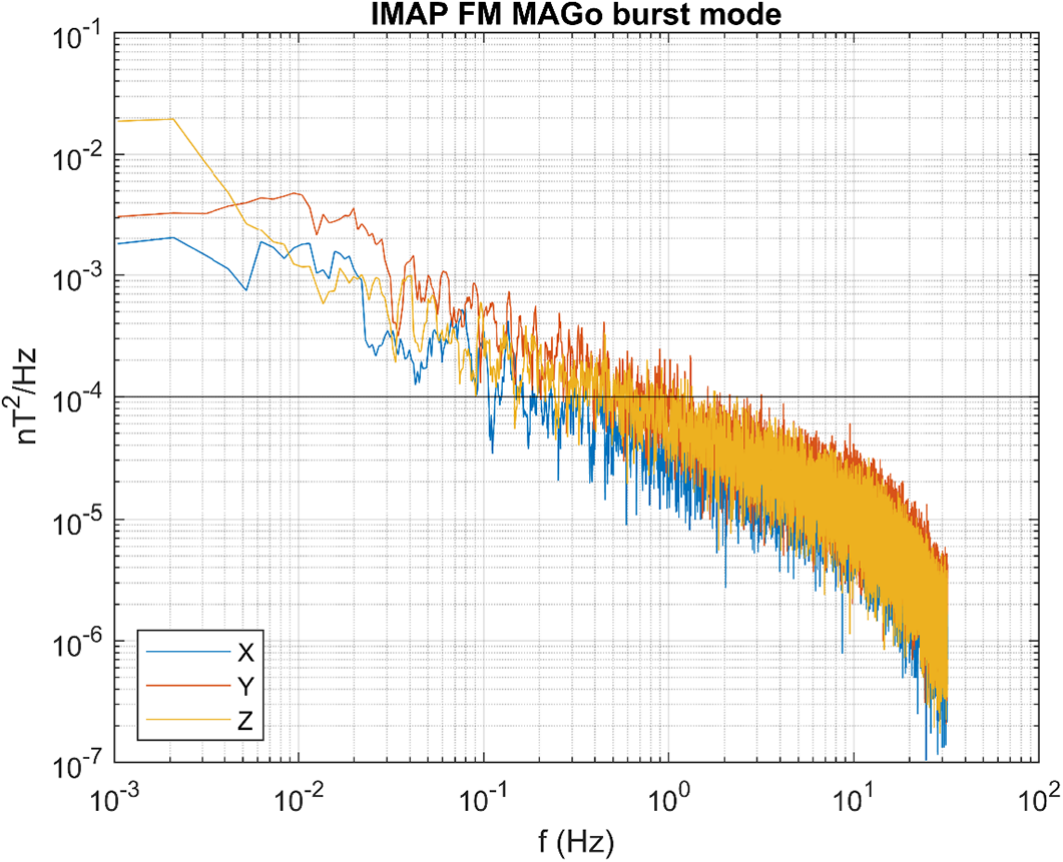
Power spectrum of MAGo flight model burst mode (64 vectors/s) data recorded inside a magnetic shielding can at Imperial College London. The horizontal line marks a level of 10 pT. The rollover above 10 Hz is due to the instrument low pass filter, as seen in Fig. 5

Noise levels for both sensors are below 10 pT at 1 Hz; an example spectrum for the 3 components of the MAGo (outboard) sensor, again taken in a shielding can at Imperial College London, is shown in Fig. 7.

Analysis has also been undertaken of the science data stream, for example by quantifying time jitter, cross-talk between sensor components, and filter performance and these are all nominal. The instrument satisfies its requirements with margin.

## Calibration

Calibration is the process by which MAG measurements are converted into scientifically useable estimates of the magnetic field at the spacecraft location. This uses ground calibration of the instrument along with in-flight estimates of spacecraft-generated magnetic fields, which can vary over a wide range of timescales.

Assuming linearity, the calibration problem can be expressed as:

$$ \boldsymbol{B}_{\boldsymbol{R}} = \boldsymbol{C B}_{S} - \boldsymbol{O} $$

Here, $\boldsymbol{B}_{R}$ is the true vector field that one wishes to estimate (in the spacecraft frame), $\boldsymbol{B}_{S}$ is that measured by the 3 axes of the magnetic field sensor, $\boldsymbol{O}$ is a vector representing stray spacecraft magnetic fields (again, in the spacecraft frame) and $\boldsymbol{C}$ is a 3x3 matrix that contains information on the gains and orientations of each sensor axis: for an ideal sensor, $\boldsymbol{C}$ would be the unit matrix, and small changes from this represent non-orthogonality of the axes and small variations in relative gains between axes. In practice, $\boldsymbol{C}$ varies little in flight, but on a spinning platform the final data product is extremely sensitive to inaccuracies in its elements, especially offsets, so it must be determined in space and updated regularly.

Variations in spacecraft-generated fields, captured in the offset vector $\boldsymbol{O}$, are typically the largest source of uncertainty in the calibrated magnetic field product; methods to estimate and remove them take advantage of particular characteristics of different spacecraft as is the case for IMAP, as described below.

### Ground Calibration

The IMAP flight model electronics and sensors were calibrated at the Magnetsrode facility near Braunschweig, Germany which has been used for many previous missions including HELIOS, Cluster, MMS, Solar Orbiter, JUICE and Europa Clipper. A 2.5 m diameter 3-axis Braunbek coil system is used to remove the Earth’s field and impose additional fields to allow instrument sensitivity, offsets, linearity, orthogonality and bandwidth to be determined. Heaters and liquid nitrogen can be used to allow parameters to be determined over a wide temperature range.

Calibration proceeded nominally and analysis of the MAG data confirmed orthogonality between axes within 1^o^, with sensitivities within ∼1% of nominal, stable within 0.5% over a temperature range of −18 °C to +21 °C. Angles were similarly stable, within ∼0.1^o^ over this range. Linearity was better than 0.5% over the realistic range of fields which will be encountered in flight.

These results confirmed the excellent performance of the instrument and its appropriateness for flight.

### In-Flight Calibration

For a spinning spacecraft such as IMAP, in-flight calibration provides more accurate estimates of some calibration parameters than can be made on the ground, so is used routinely to determine sensor alignment angles, relative gains and spacecraft-generated magnetic fields. Imperial College London has nearly 25 years’ experience calibrating the magnetometers on the four Cluster spacecraft (Balogh et al. 2001) and while a new processing pipeline has been developed for IMAP, the same fundamental calibration principles are used for IMAP as have been applied to Cluster data. These are largely based on the Kepko et al. (1996) method to determine most calibration parameters such as alignment angles and relative gains, but the spin axis offset remains a challenging quantity to estimate. Historically, a number of methods have been developed (e.g. Hedgecock 1975) but for IMAP, an evolution of the Leinweber et al. (2008) method for solar wind data is used.

IMAP launches on the same vehicle as NOAA’s Space Weather Follow-On L1 mission (SWFO-L1), which also carries a magnetometer (Torbert et al. 2026), and travels on a similar trajectory towards L1. There is the opportunity to compare data from the two instruments during their cruise to L1: SWFO-L1 is a 3-axis stabilised spacecraft and has a 6 m boom, so the inter-calibration is likely to be useful for both instrument teams. We expect that there will also be opportunities to compare IMAP data with that from near-Earth missions in the solar wind such as THEMIS, as well as those at L1 such as ACE, Wind, DSCOVR and Aditya-L1.

### Magnetic Cleanliness

Critical to the success of the calibration process is the minimisation of artificial fields, for which an extensive Electromagnetic Environment Control Program was implemented, led by APL. The overall mission-level magnetic cleanliness requirements were flowed down to instrument and subsystem level for both DC and AC fields. Key subsystems such as the solar arrays were treated at design level, while others such as instrument units were characterised at both EM and FM stage to ensure compliance with the overall magnetic budget. Magnetic screening of all subsystems was undertaken before integration.

A DC mathematical magnetic model of the spacecraft was constructed at APL using subsystem measurements, and verified using unpowered spacecraft-level testing in November 2024 after integration of almost all units. This was not a swing test; rather, the spacecraft was suspended from a hoist and moved to a pre-determined set of distances from an array of magnetometer sensors, with an orientation such that the field along the deployed boom direction was measured. Results were consistent with the model and well within the requirement of <20 nT fields at the MAGo deployed location. This test was repeated successfully for the final flight configuration of the spacecraft at Astrotech Space Operations before encapsulation.

Time-varying fields are of generally greater concern than DC, since they are harder to characterise and remove in the calibration process. Again, subsystems have been characterised during operation and current consumption profiles measured, since stray fields can often be due to current loops rather than magnetic material: knowledge of typical current variations and how they vary during particular operations helps to inform possible sources of noise.

Some spacecraft activities inevitably produce a magnetic signal. Thruster firings are the largest source: knowledge of their operation times, combined with the application of gradiometer analysis, makes it possible to remove these signals using in-flight calibration. Another culprit is the IMAP-Lo pivot platform (Schwadron et al. 2025) which is expected to move several times a day. This unit has been extensively characterised on the ground, including fields from the platform at various orientations, and these measurements will be used to inform the in-flight cleaning procedures using housekeeping and commanding information to determine the pivot platform location as a function of time.

In practice, early flight data shows the spacecraft overall to be magnetically very clean, with few signals in the data beyond those associated with thruster firings and pivot platform movements. Automated thruster and pivot platform removal algorithms are under development and will be applied to the science-quality, Level 2 data as described in the Data Products and Algorithms section.

The sensitivity of MAG measurements to signals at the 2$f$ sense frequency resulted in an additional keep-out requirement at 30.720 kHz ±100 Hz and 61.440 kHz±60 Hz, verified by design and EMC testing.

## Operations

Operation of MAG has been planned to be as simple as possible while still achieving the science objectives. MAG operates continuously after commissioning, through the cruise phase and into the primary science phase of the mission. MAG has two science operating modes: Normal and Burst. The cadence of these can be altered in flight (see Table 6) but the nominal operations, given the telemetry budget, are for 2 vector/s to be recorded from each sensor in Normal mode and 64/s from the outboard and 8/s from the inboard during Burst mode.

**Table 6:** MAG operating modes

Mode	Primary vectors/s	Secondary vectors/s	Default packet cadence	Comment
N_2_2	2	2	32 s	Default normal mode
N_4_1	4	1	8 s	
N_4_4	4	4	8 s	
B_64_8	64	8	4 s	Default burst mode
B_64_64	64	64	4 s	
B_128_128	128	128	2 s	
I-ALiRT	1/4	1/4	1 s	Real time space weather data – data sent over 4 packets

An additional benefit of the onboard data compression scheme is that the number of bits to be transmitted can be varied. Without compression, 16 bits are used for each component: for a given resolution (size of the lowest bit, in nT) this limits the maximum value that can be measured. This drives the need for autoranging, where if the field approaches the maximum value in one range, the instrument automatically moves into a range which can cover a larger range of field values, but due to the fixed number of bits returns data at a lower resolution. The compression scheme can be configured to use more bits and nominal operation will be to use 18: this results in a maximal precision operation while accommodating a larger overall field before ranging is required. For IMAP, MAG will run with 4.5 pT precision and a range of ±512 nT, meaning that the instrument is unlikely to need to range at any time unless an extreme Carrington-like coronal mass ejection passes over the spacecraft. Autoranging is still enabled within compression, so the instrument will change range in this circumstance as required to continue making science measurements in a large field. In this case, the instrument will automatically drop back to the most sensitive range when the field remains below the transition level for a time which is initially configured at 20 s, above the spin period, to ensure that rapid range changes do not occur as the spacecraft rotates. It is possible to force the instrument into a large range even in the presence of small fields, at which point it will auto-range back to the most appropriate. This operation makes it possible to determine the relative offsets of all the instrument ranges: this will be commanded several times a year to maintain full offset knowledge.

Real-time I-ALiRT data is generated at the rate of one inboard and one outboard vector every 4 seconds. These vectors are split over four packets, which are transmitted at 1 s cadence. Transmission of both inboard and outboard data allows automated gradiometer methods to be used to clean the data.

In addition to magnetic field measurements, MAG generates comprehensive housekeeping data to allow monitoring of instrument health: the Payload Operations Center can respond to out of limit measurements for several parameters including temperatures and voltages. In addition, for some limit violations the instrument will generate an event packet in real time, in response to which the spacecraft will power it down. Within the I-ALiRT packets a small amount of housekeeping data such as key voltages and temperatures is also transmitted, making it possible for the MAG team to monitor instrument health even outside science telemetry passes.

### Early Operations and Commissioning

Magnetometers on science missions are usually carried on long booms to place them as far as possible from magnetic fields generated by the spacecraft and IMAP is no exception, with a single hinged rigid 2.5 m boom. In this case, it is helpful to take magnetic field measurements during boom deploy: data before, during and after deployment make it possible to better characterise the spacecraft fields and verify the spacecraft magnetic model. An extended boom helps to stabilise a spinning spacecraft such as IMAP and so deployment occurs three days after launch when the spacecraft is in the solar wind upstream of Earth.

Following deployment, MAG is powered off for several hours to allow the sensors to cool, after which instrument commissioning occurs. This is a relatively straightforward procedure, with all the instrument modes being exercised and the first data taken to allow initial in-flight calibration to begin.

MAG remains powered throughout the extended commissioning sequence of the rest of the payload, taking normal mode data except while each instrument commissions, when 128 vector/s burst mode data is taken on both sensors to provide a characterisation of the magnetic signatures of the units and improve cleaning of data in the science phase.

### Science Operations

MAG operates and takes science measurements continuously during the science phase of the mission. While MAG can operate at a number of different cadences in normal and burst mode, summarised in Table 6, in practice, it is expected that MAG will record vectors at 2 vectors/s from both sensors in normal mode, while taking 64 vectors/s from the outboard sensor and 8 vectors/s from the inboard in burst mode. This provides an optimal combination of high cadence to access ion kinetic scales, while also providing inboard sensor data at a rate sufficient to clean the outboard data of spacecraft interference signals. While the instrument can operate at 128 vectors/s, this is not expected to be used after commissioning due to the large data volume generated, and that fact that there is rarely a physical signal above the instrument noise floor at frequencies above 32 Hz.

In common with other instruments, MAG operations are as simple as possible: MAG remains in normal mode for most of each day and is commanded into burst mode once per day, with a duration that is commensurate with the downlink budget. Given the expected performance of the compression algorithm, this is likely to be around 8 hours per day, but this will be refined during the cruise phase and if more telemetry is available it could be extended and even run continuously. The time of the burst mode command each day will be determined by the Payload Operations Center (POC) to minimise overlap with ground passes and movements of the IMAP-Lo pivot platform because magnetic interference is expected to be higher during these times.

MAG’s real-time I-AliRT data is transmitted continuously by the spacecraft: when ground station coverage is available the data is downloaded and transmitted to the Science Data Center (SDC; Reno et al. 2026) where it is processed and made publicly available (Lee et al. 2025).

## Data Products and Algorithms

The goal of the MAG team is to provide simple, easily-useable high quality data products in a timely manner. Key data products of interest to the science and space weather community are summarised in Table 7.

**Table 7:** Top level MAG data products

Mode	Vectors/s	Coverage	Coordinates
Normal	2	Continuous	RTN, GSE, GSM
Burst	64	∼8 hours/day	RTN, GSE, GSM
I-ALiRT	0.25	Continuous, real-time	GSE, GSM

In common with the other IMAP instruments, MAG data are processed at the Science Data Center (SDC) using algorithms developed and agreed between the instrument team and SDC. Upon ground receipt, the data is processed to several levels, as described in Table 8. Since the MAG instrument does not produce normal mode data when burst mode is selected, Level 1c processing is used to produce filtered, downsampled data at the normal mode cadence from the burst mode stream, ensuring a continuous, statistically homogeneous normal mode stream throughout the mission.

**Table 8:** MAG science data products at Level 0, 1 and 2

Level	Description
Level 0	Data in CCSDS packets in engineering units
Level 1a	Data separated into streams for each sensor, burst and normal mode
Level 1b	Transformed into sensor unit reference frame; fixed time shift applied
Level 1c	Continuous normal mode data, generated using filtered downsampled burst mode data when necessary
Level 1d	Preliminary, automated calibrated data for rapid use by IMAP instrument teams. Not full science quality
Level 2	Science quality normal (continuous) and burst (intermittent) mode data. GSE, GSM and RTN coordinates.

Data at levels 0 to 1d are generated automatically at the SDC. Level 2 data can only be produced once detailed calibration files have been generated by the MAG team at Imperial College London. This process takes time, using the methods described earlier in this paper and this was the motivation for the definition of the Level 1d product, which will provide team members early access to a cleaner product than Level 1c by removing running hourly spin plane averaged fields, a pre-calculated spin axis offset and using a simple gradiometer algorithm to clean the largest spacecraft signals. Other cleaning, for example of the IMAP-Lo pivot platform movement, is not explicitly removed for Level 1d.

MAG Level 2 data products are the highest quality estimates of the magnetic field at the spacecraft location and are what should be used for science analysis. They are publicly released along with other IMAP products, 3 months after receipt on the ground.

The I-ALiRT real-time data are released immediately on receipt and are cleaned with a similar algorithm to Level 1d data; this produces a product which is usable for space weather forecasting but not science analysis.

## Conclusions

MAG satisfies all its science requirements and contributes to both key science goals of the mission and real-time space weather data. Following launch on 24 September 2025, MAG has completed commissioning and is functioning well in flight.
